# Harnessing in Silico Design for Electrochemical Aptasensor Optimization: Detection of Okadaic Acid (OA)

**DOI:** 10.3390/bios15100665

**Published:** 2025-10-03

**Authors:** Margherita Vit, Sondes Ben-Aissa, Alfredo Rondinella, Lorenzo Fedrizzi, Sabina Susmel

**Affiliations:** 1 Bioanalytical Chemistry and Biosensors Lab, Department of Agri-Food, Environment and Animal Sciences (Di4A), University of Udine, Via Sondrio 2/A, 33100 Udine, Italy; margherita.vit@uniud.it; 2 Molecular Sciences Research Hub, Department of Chemistry, Imperial College London, London W12 0BZ, UK; s.ben-aissa@imperial.ac.uk; 3 Polytechnic Department of Engineering and Architecture, University of Udine, Via del Cotonifico 108, 33100 Udine, Italy; alfredo.rondinella@uniud.it (A.R.); lorenzo.fedrizzi@uniud.it (L.F.)

**Keywords:** okadaic acid, electrochemical aptasensor, in silico modeling, aptamer truncation, probe design, ferrocene label

## Abstract

The urgent need for advanced analytical tools for environmental monitoring and food safety drives the development of novel biosensing approaches and solutions. A computationally driven workflow for the development of a rapid electrochemical aptasensor for okadaic acid (OA), a critical marine biotoxin, is reported. The core of this strategy is a rational design process, where in silico modeling was employed to optimize the biological recognition element. A 63-nucleotide aptamer was successfully truncated to a highly efficient 31-nucleotide variant. Molecular docking simulations confirmed the high binding affinity of the minimized aptamer and guided the design of the surface immobilization chemistry to ensure robust performance. The fabricated sensor, which utilizes a ferrocene-labeled aptamer, delivered a sensitive response with a detection limit of 2.5 nM (*n* = 5) over a linear range of 5–200 nM. A significant advantage for practical applications is the remarkably short assay time of 5 min. The sensor’s applicability was successfully validated in complex food matrices, achieving excellent recovery rates of 82–103% in spiked mussel samples. This study establishes an integrated computational–experimental methodology that streamlines the development of high-performance biosensors for critical food safety and environmental monitoring challenges.

## 1. Introduction

**Figure 1:**
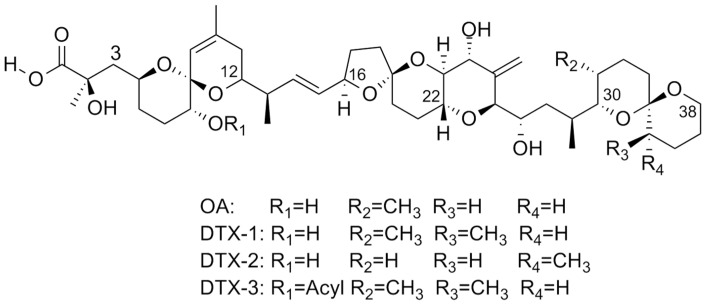
Structure of okadaic acid (OA) and of the parent toxins, dinophysistoxins (DTXs).

Harmful Algal blooms (HABs) are associated with the production of marine biotoxins, among which okadaic acid (OA) and its analogs (Figure 1), dinophysistoxins (DTXs), are of significant concern. These lipophilic toxins accumulate in filter-feeding bivalves, such as mussels, and are responsible for diarrhetic shellfish poisoning (DSP) in humans, a gastrointestinal syndrome marked by acute symptoms including diarrhea, nausea, and vomiting. Recent reports also highlight the increasing incidence of HABs in historically low-risk environments, particularly in Mediterranean coastal areas, Adriatic aquaculture zones, and temperate inland waters, where such events now lead to ecological degradation and socio-economic negative effects for tourism and fisheries, and have induced harvesting bans [1,2]. HAB-related toxins significantly impact global aquaculture economies, with annual losses exceeding USD 8 billion [3]. Notably, the Copernicus Ocean State Report 8 (2024) describes an exceptional phytoplankton bloom in the southeastern Mediterranean (south of Crete), with a 50% increase in spatial extent and a 35% rise in primary productivity, illustrating the shifting dynamics and growing unpredictability of algal bloom patterns ([4]). From this scenario it emerges that an accurate and timely detection of OA and its congeners is therefore critical for food safety assurance and environmental monitoring.

Currently, official monitoring programs rely predominantly on instrumental analytical methods such as high-performance liquid chromatography (HPLC) and liquid chromatography coupled with tandem mass spectrometry (LC-MS/MS). These techniques offer exceptional sensitivity and specificity, achieving detection limits as low as 3 µg/kg and quantification limits of 10 µg/kg in shellfish matrices [5], meeting regulatory requirements such as the Codex Alimentarius threshold of 160 µg/kg (198 nM) [6]. In addition to these instrumental approaches, immunoassays such as enzyme-linked immunosorbent assays (ELISAs) have been explored as alternative methods. ELISA offers high specificity and sensitivity in the micro and nanogram-per-milliliter range [7,8], yet its implementation remains labor-intensive, requiring multiple incubation and washing steps, and substantial sample volumes, and is prone to operator-induced variability [9]. Similarly, phosphatase-based assays have demonstrated compliance with regulatory detection limits [10], but involve complex multistep protocols, including pre-labeling of substrates, making routine monitoring challenging, particularly in resource-limited settings [11,12].

Biosensors have emerged as promising alternatives for marine toxin detection, offering rapid analysis, cost-effectiveness, and potential for field deployment. These systems have been widely utilized for the detection of okadaic acid (OA), leveraging the high affinity and specificity of antigen–antibody interactions [13,14,15,16]. Specifically, those employing optical transducers for signal generation have shown promising performances [17,18,19,20]. For instance, an optical biosensor immunoassay achieved an action limit of 120 µg/kg and a detection limit of 31 µg/kg for OA in shellfish, with a working range of 31–174 µg/kg [18]. Another competitive amplified luminescent proximity homogeneous assay (AlphaLISA) demonstrated a detection range of 0.02–200 ng/mL with a detection limit of 4.55 × 10^−3^ ng/mL within 15 min [21]. Electrochemical immunosensors have also shown notable sensitivity. A graphene-based voltammetric immunosensor achieved a detection limit of 19 ng/L for OA in PBS buffer [14]. Another label-free impedimetric immunosensor reported a linear range of 0.195–12.5 µg/L with a detection limit of 0.3 µg/L for OA in mussel samples. More recently, a highly sensitive electrochemical immunosensor using carbon black modified screen-printed electrodes reported a detection limit of 0.15 ng/mL for OA in buffer and 0.18 ng/mL in mussel extract [22]. Nevertheless, despite their proven effectiveness, immunosensors possess several inherent limitations that hinder their wider adoption, particularly in resource-limited settings. The production of highly specific antibodies is both time-consuming and costly, often necessitating animal immunization protocols, which raises ethical concerns and complicates reproducibility and large-scale manufacturing. Moreover, antibodies are susceptible to denaturation or loss of function under non-physiological conditions, such as elevated temperatures, organic solvents, or extreme pH values ([14,15,23]). This can lead to poor storage stability, reduced shelf life, and performance variability between production batches, all of which compromise the reproducibility and robustness of the final sensing device. Therefore, to overcome the limitations of antibody-based systems (e.g., cost, stability, and ethical aspects), alternative biorecognition elements have been increasingly investigated in recent years. Among these, aptamers have emerged as a particularly promising option. Aptamer-based biosensors (aptasensors) offer distinct advantages. Aptamers are synthetic single-stranded oligonucleotides that can fold into complex three-dimensional conformations through intramolecular base pairing [24,25]. These conformations allow aptamers to bind a wide variety of targets, including small molecules, proteins, and whole cells, with high affinity and specificity [26]. Compared to antibodies, aptamers offer several key advantages: they are chemically synthesized, eliminating batch-to-batch variability and the need for animal-derived components, while also offering enhanced thermal and chemical stability, longer shelf life, and ease of modification [27,28,29]. When incorporated into biosensors, commonly termed aptasensors, aptamers enable highly sensitive and selective detection of target analytes, often using electrochemical (i.e., EAB) or optical transduction mechanisms [27].

The selection of aptamers is performed using SELEX (Systematic Evolution of Ligands by EXponential enrichment), which remains the fundamental method for initial aptamer discovery through its iterative process of binding, separation, and amplification [24,25]. While SELEX is effective, it is often time-consuming, requiring weeks or even months, and can yield sequences with limited specificity due to off-target binding or the formation of undesired secondary structures [30,31]. To enhance SELEX-selected aptamers, computational approaches have emerged as valuable complementary tools for post-SELEX optimization. For instance, molecular docking simulates aptamer–target interactions in three-dimensional space using predictive algorithms, allowing for rapid screening of large sequence libraries and providing mechanistic insights into binding affinity and selectivity [32,33]. This computational optimization approach, when applied to previously SELEX-selected sequences, enables targeted improvements via truncations or mutations while maintaining the essential binding properties established through the original selection process. The aptamer’s structural flexibility enables formation of essential dynamic motifs (stem-loops, hairpins, and G-quadruplexes) for target recognition [34,35], but its length presents challenges including non-specific interactions, and increased baseline noise has been reported. Therefore, in silico simulation-assisted truncation aims to preserve the functional binding domains while minimizing unnecessary nucleotides, thus enhancing affinity and simplifying chemical synthesis [32,36,37,38]. Importantly, molecular docking was not only employed to enhance binding affinity, but also to guide the rational selection of aptamer regions most involved in target-specific interactions, thereby contributing to the improvement of selectivity. This approach has been successfully used in previous studies aiming to optimize aptamer sequences to improve its affinity to analytes [36,39,40,41]. However, if it provides valuable insights for aptamer optimization, these computational predictions require experimental validation to confirm their accuracy in real conditions.

For instance, an electrochemical aptasensor for OA demonstrated a linear range between 100 pg/mL and 60 ng/mL with a detection limit of 70 pg/mL ([42]). Another multiplexed electrochemical aptasensor reported a detection limit of 0.0048 nM for OA [43]. This study utilized a patented 63-nucleotide aptamer (EP2770058A1), characterized by Marty & Prieto-Simón [44], as the foundation for computational optimization, aiming to identify the specific sequence and to drive the truncation. After that, we used the in silico approach to design the complementary capture probes of varying lengths for aptamer immobilization on electrode surfaces and to estimate the binding of both OA63 and new-OA31 Apts to OA and its structural analogs (i.e., DTXs). To corroborate the findings of the molecular docking, several experimental trials for functioning assessments were carried out and the EAB was optimized. The resulting sensor, incorporating thiol-functionalized capture probes and ferrocene-tagged aptamers (at either 3′ or 5′ end), detected OA within 5–200 nM, with a detection limit of 2.5 nM (=3 s of blank signal) using an incubation time of 5 min at 4 °C. The sensor allowed a very fast detection of toxins in spiked mussel samples with recovery rates in the range of 82–103%. Our experimental results corroborated the computational findings, confirming the detection of all toxins—an outcome that, while indicative of limited selectivity to OA, is nonetheless valuable given their shared toxicological effects. Both OA63 and OA31 aptamers successfully detected OA under optimized conditions, demonstrating the adaptability of aptamer-based systems. The correlation between computational predictions and experimental results demonstrates the utility of rational in silico design for optimizing electrochemical aptasensors for marine toxin detection, offering a systematic approach to biosensor development.

## 2. Experimental Section

### 2.1. Materials and Methods

#### 2.1.1. Materials and Reagents

Materials and reagents were obtained from commercial sources and used as received. Two ferrocene (Fc)-tagged aptamers and four thiol-modified capture probes were synthesized by Sigma (IT) upon request (Table 1). Chemical reagents including 1,4-dithiothreitol (DTT), magnesium chloride, trizma hydrochloride (Tris), tetra-chloroauric gold, tris(2-carboxyethyl)phosphine (TCEP), 6-mercaptohexanol (MCH), and nuclease-free water were purchased from Sigma Aldrich (IT). Okadaic acid (OA) and dinophysis toxins (DTX1 and DTX2) were obtained from CIFGA (Spain). Stock solutions were prepared in sterile nuclease-free water under a PCR-grade fume hood (Bioscientifica–model PCR UVC/T-M-AR): aptamers and capture probes as 100 μL aliquots at 100 μM, OA at 9.63 μM, and DTT at 400 μM in MilliQ water. All solutions were stored at −20 °C until use, with OA was kept in dark conditions. Assays were conducted in Tris Buffer (50 mM Tris, 150 mM NaCl, 2 mM Mg ^2+^, pH 7.5).

#### 2.1.2. Instrumentation

Electrochemical experiments were performed with a CHI 730 model of a potentiostat/galvanostat (CH Instruments Inc., Bee Cave, Texas, USA). Amperometry, Cyclic Voltammetry (CV), and Differential Pulse Voltammetry (DPV) were adopted by applying parameters purposely optimized as follows: initial potential –0.5 V, final potential +0.6 V, increment 0.004 V, pulse amplitude 0.05 V, pulse width 0.025 s, sample width 0.02 s, pulse period 0.5 s, sensitivity 1 × 10^−4^ A/V, and scan rate 100 mV/s (see Table S1). Gel Electrophoresis was performed with PowerPac Basic Power Supply (Bio-Rad Laboratories, Inc. Italy). Scanning electron micrographs (SEMs) were collected with a JEOL JCM-7000 emission scanning electron microscope. An accelerating voltage of 5.0 KV was used and the images were analyzed with a magnification of 250×, 1000×, 2500×, 5000×.

### 2.2. Computational Docking

Computational analysis employed a suite of web-based and standalone software tools (UNAFOLD 3.6 version, RNAComposer freely available at http://rnacomposer.ibch.poznan.pl and http://rnacomposer.cs.put.poznan.pl), Discovery Studio Visualizer 2023 version, and AutoDock Vina 1.2.3 version) to generate 3D structures of the aptamers and OA target for docking simulations. Four capture probes were designed in silico for aptamer immobilization: two shorter (6 and 8 bases) and two longer sequences (14 and 16 bases). These probes were designed to hybridize with the non-labeled end of each aptamer and were functionalized with thiol groups for attachment to gold-modified electrodes. The aptamer sequences (OA63 and OA31) were tagged with ferrocene (Fc) as the electrochemical reporter. The complete sequences and specifications are provided in Table 1.

### 2.3. E-Aptasensor Fabrication and Optimization

#### 2.3.1. Gold Deposition on Screen Printed Electrodes (Au@SPCE)

Commercial Screen-Printed Carbon graphite Electrodes (SPCE; Ecobioservices, Sesto F.no (FI), Italy) were modified with gold using a two-step electrochemical process. The working electrode (WE) surface was first activated by chronoamperometry (CA) in 1 M NaHCO_3_ at −1.3 V for 5 min. After chronoamperometric pre-treatment in 1 M NaHCO_3_ (−1.3 V, 5 min), the electrode was rinsed with ultrapure water (3 × 200 µL) to remove residual bicarbonate buffer. Gold deposition was then performed by dropping 50 μL of AuHCl_4_ (5 mM in 0.1 M KCl) onto the WE and applying CA at −0.4 V for 1200 s. The resulting gold layer thickness, calculated from the charge transfer (see Supplementary Materials Paragraph S4), was 26.54 ng (RSD ± 0.25%, n = 30).

#### 2.3.2. Aptamer Immobilization at the Electrode Surface

Two distinct protocols were developed and compared for aptamer immobilization on the gold-modified electrodes.

**Scheme 1:**
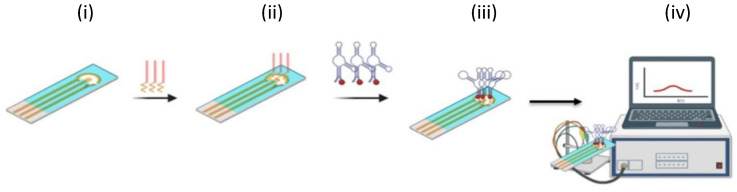
Fabrication strategies for the E-aptasensor. (A) Protocol 1—heterogeneous approach: (i) Au electrodeposition on SPCE; (ii) thiolated capture probe immobilization; (iii) aptamer hybridization on the electrode surface; (iv) DPV readout (ferrocene redox signal).

In Protocol 1 (heterogeneous approach, Scheme 1), after Au electrodeposition (i), the thiol-modified capture probe (5 μL, 2 μM) was mixed with DTT (2.5:25 μM ratio) and immediately deposited on the Au@SPCE electrode surface for overnight chemisorption at room temperature (ii). Subsequently, the aptamer solution (5 μL, 5 μM in Tris buffer) was introduced and incubated for 3 h at room temperature (iii). The electrode was then washed with Tris buffer (50 μL, 0.1 M) prior to analysis and DPV signal was recorded (iv).

**Scheme 2:**
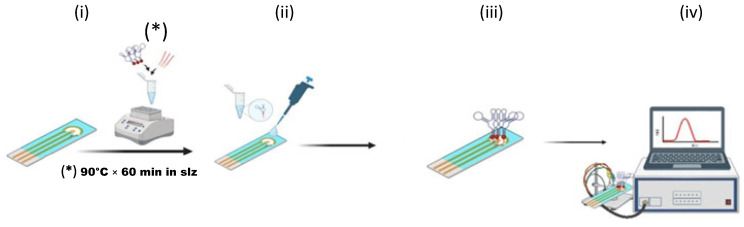
Fabrication strategies for the E-Aptasensor. (B) Protocol 2—homogeneous approach: (i) hybridization of capture probe and aptamer in solution; (ii) deposition of the pre-formed complex onto Au@SPCE; (iii) electrode blocking and stabilization; (iv) DPV readout (ferrocene redox signal).

Protocol 2 (homogeneous approach, Scheme 2) involved a hybridization step of the capture probe and aptamer (2.5:5 μM ratio, respectively) carried out in solution made of 30 μL Tris buffer. The optimal time of incubation carried out at 90 °C in a thermal block was identified as 60 min after testing 10 and 30 min as well (ii). After the hybridization in solution, the protocol was similar to Protocol 1; in fact, a volume of 5 μL of this solution was placed on the electrode surface already prepared to obtain an Au@SPCE (i) and it was incubated overnight at 45 °C in the presence of DTT (25 μM) (iii). The modified electrode was washed with Tris buffer (50 μL, 0.1 M) before electrochemical measurements (iv).

Both protocols, namely 1 and 2, were systematically evaluated using the possible “capture probe-aptamer” combinations, i.e., shortest and longer capture probe P63_8, P63_16 were both tested with OA63Apt and shortest and longer capture probe P31_6, P31_14 with OA31Apt.

### 2.4. Evaluation of E-Aptasensor Performance for OA Detection In Vitro

The E-aptasensor response to OA was evaluated across a concentration range of 5–200 nM. Measurements were performed by depositing 50 µL of OA solution onto the electrode surface, aptamer-modified. All measurements were carried out in Tris buffer (50 mM Tris, 150 mM NaCl, 2 mM Mg^2+^, pH 7.5). Each experiment was using 5 independent electrodes (n = 5). Binding kinetics for both OA63 and OA31 aptamers were assessed at 25 °C and 4 °C, with interaction times of 5, 30, and 60 min. The same protocol was employed to evaluate cross-reactivity with DTX1.

### 2.5. Evaluation of E-Aptasensor Performance for OA Detection in Real Samples

Preparation of real mussel samples for OA analysis was performed following a modified protocol from Zeng et al. [45]. Briefly, 2 g of shellfish meat, sourced from a local market and previously verified as safe for human consumption by relevant national food safety authorities, underwent sequential extraction with methanol (9 mL) through a two-step process. Each extraction involved 60-s mixing followed by centrifugation (8000× *g* rpm, 5 min). The combined supernatants were evaporated under nitrogen to near-dryness and reconstituted in Tris buffer (1 mL) for aptasensor analysis.

For recovery studies, commercial mussels were spiked with OA at concentrations ranging from 5 to 200 nM, including the regulatory limit 160 µg/kg (equivalent to 198 nM). Spiked samples were incubated overnight at 4 °C before extraction and analysis. This concentration range aligned with both the sensor’s analytical working range and regulatory requirements for OA in edible mussels.

## 3. Results and Discussion

### 3.1. Computational Analysis and Aptamer Design

A suite of web-based computational tools was employed to generate the three-dimensional (3D) structures of the molecular partners—namely, the aptamers and the okadaic acid (OA) target—as a prerequisite for molecular docking simulations.

**Figure 2:**
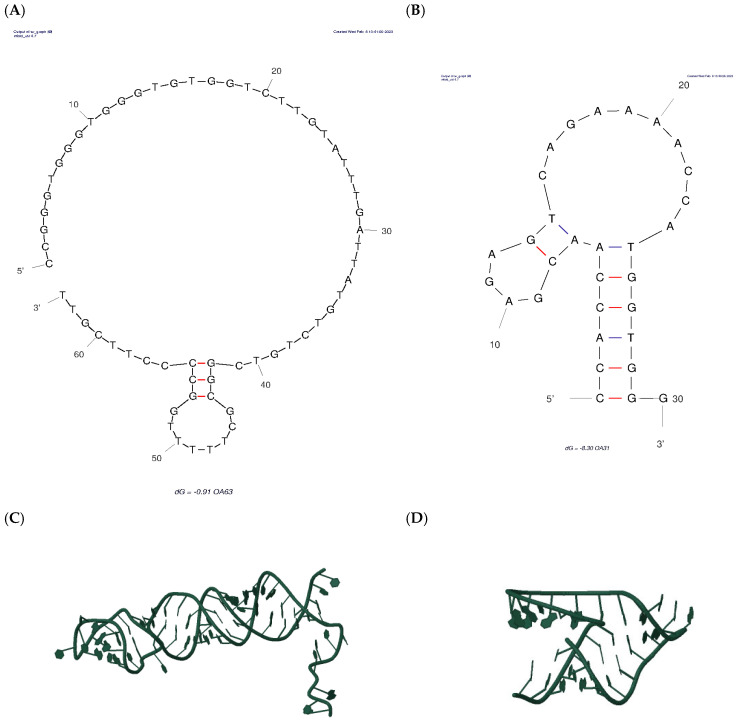
2D and 3D structures of the OA63 apt (**A**,**C**), the truncated OA31 apt (**B**,**D**). (Red Lines: less stabile interactions).

Initially, the full-length 63-nucleotide aptamer sequence (EP2770058A1) was processed through UNAFOLD to generate its two-dimensional (2D) structure under experimentally relevant conditions of temperature and ionic strength (Figure 2A). The structural modeling proceeded through a multi-step process: (i) conversion of the 2D single-stranded DNA structure to a 3D RNA model using RNAComposer (Figure 2C), including necessary nucleotide modifications; (ii) transformation back to DNA format via reverse mutation of uracil (U) to thymine (T) using x3DNA.org web tool; and (iii) refinement of sugar moieties using Discovery Studio Visualizer. The 3D structure of OA was obtained from PubChem database.

Upon obtaining the 3D coordinates for both the 63-nucleotide aptamer and OA, molecular docking was carried out using AutoDock Vina, designating the aptamer as the receptor and OA as the ligand. The in silico approach was used to identify the nucleobases in the 63-apt interacting with the OA before the resizing of the apt itself. So, insights from the docking simulations guided the rational truncation of the original aptamer to a 31-nucleotide version, preserving the binding site to potentially improve affinity. The truncated new aptamer underwent the same 3D modeling pipeline, and its structure was subsequently used in docking simulations for OA binding (Figure 2B,D) [39,46].

Computational analysis with UNAFOLD revealed that the OA63 aptamer’s binding pocket for Ochratoxin A (OA) resides within its first 31–34 nucleotides, which fold into a stable stem-loop structure. Such motifs are fundamental to aptamer performance: the loop presents the specific nucleotides for target recognition, and the stem contributes thermodynamic stability and fine-tunes affinity [47,48,49]. Accordingly, the aptamer was rationally truncated to 31 bases, a strategy designed to isolate the recognition domain and remove non-essential residues. This truncation minimizes the likelihood of alternative secondary structures, a common post-SELEX refinement technique used to enhance structural uniformity and binding performance [50]. Recognizing that sequence shortening and surface immobilization can restrict an aptamer’s conformational flexibility, capture probes of different lengths were also developed. These probes, detailed in Table 1 (Section 2.2), were designed from secondary structure predictions and included short (6–8 nucleotides) and long (14–16 nucleotides) versions. The longer probes were engineered with a poly-thymine (polyT) spacer at their thiolated end. This design allows for the investigation of duplex stability and the influence of aptamer mobility on competitive OA binding at the electrode surface [51,52,53,54].

**Figure 3:**
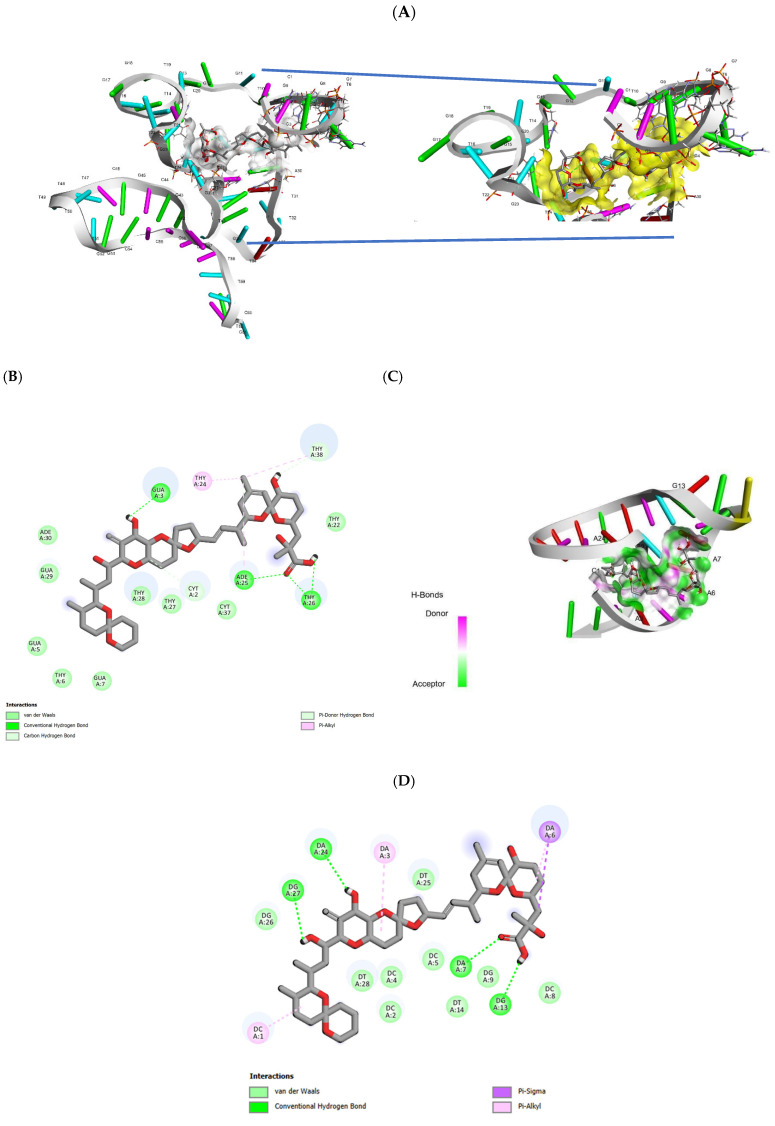
Docking investigation of the bases interacting with okadaic acid (OA) in the patented OA63apt (**A**) and (**C**) OA31 apt, here prepared by truncation, and the moieties interacting on the side of OA (**B**,**D**).

In the complex “OA63Apt-OA”, the lowest energy conformation (Figure 3A) exhibited multiple stabilizing interactions: hydrogen bonds formed between the OA carboxyl group (C1) and nucleotides Thy-26 and Ade-25, while additional hydrogen bonding occurred between hydroxyl groups (C7, C24) and nucleotides Thy-38 and Gua-3, respectively. The complex was further stabilized by π-alkyl interactions between OA’s C10 and nucleotides Ade-25, T-24, and Thy-38, along with van der Waals interactions involving the C7 hydroxyl group and Thy-38. The calculated Gibbs free energy (ΔG = –10.1 kcal/mol) indicated strong binding affinity [39,46,55]).

Critical analysis of the binding interface revealed that the majority of OA-interacting nucleobases resided within the first 31–34 residues, with the binding site corresponding to a stem-loop structure formed by Gua-3 and Thy-24 (Figure 3A). This observation provided the structural basis for rational truncation at Thy-32, generating the shorter OA31 variant. The truncated sequence underwent identical structural modeling and docking analysis (Figure 2B,D), maintaining the key binding elements while reducing potential non-specific interactions.

Molecular docking simulations of the truncated OA31 aptamer (Figure 3C) revealed binding interactions comparable to OA63, though involving different nucleobases. The OA C1 carboxyl group formed hydrogen bonds with Gua-13 and Ade-7, while hydroxyl groups at C24 and C27 established hydrogen bonds with Ade-24 and Gua-27, respectively. Additional stabilization occurred through π-alkyl interactions between C3/C5 and Ade-6, C21-C22 and Ade-3, and C32 with Cyt-1, supplemented by van der Waals forces. The calculated Gibbs free energy (ΔG = –11.7 kcal/mol) surpassed that of OA63, confirming the retention of high-affinity binding in the truncated sequence. Finally, using these findings, both aptamers were synthetized to carry out the experimental optimization, bearing the Fc electroactive tag at the 5′ in OA63 and at 3′ in OA31 (Table 1) to perform an electrochemical direct measurement.

### 3.2. Development and Characterization of Au@SCPE Platform

**Figure 4:**
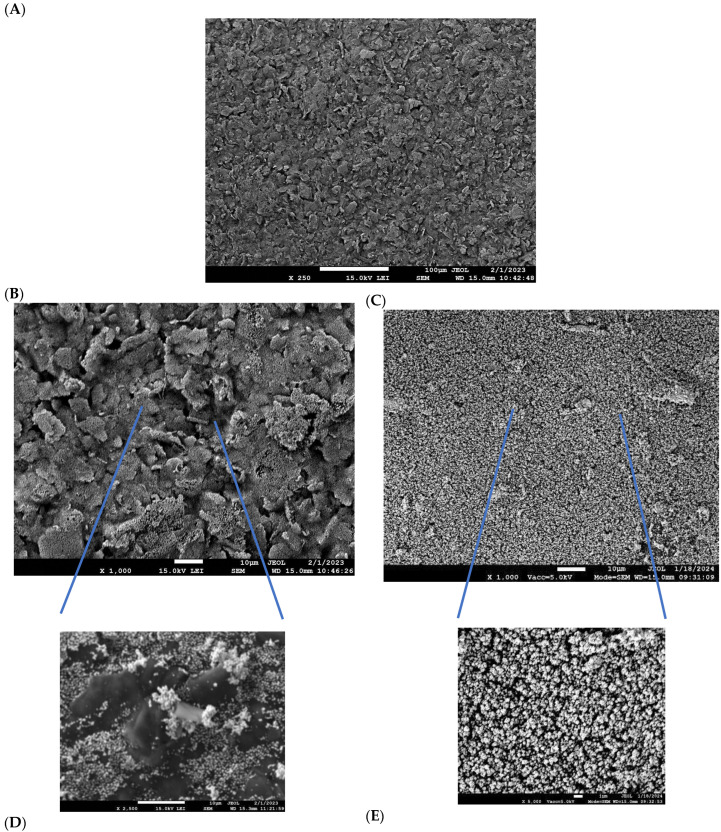
SEM of (**A**) pristine graphite electrode; (**B**) Au@SPCE without and (**C**) after pre-treatment at a constant voltage −1.3 V, 5 min in 1 M NaHCO_3_; (**D**) zoom-in of (**B**), (**E**) zoom-in of (**C**). Same magnifications are compared.

The aptasensor platform was constructed through electrochemical modification of graphite screen-printed electrodes. The pre-treatment step, consisting of applying −1.3 V for 5 min in 1 M NaHCO_3_, enhanced the surface polarity through hydroxyl (-OH) and carboxylate (-COO^−^) group formation. This process facilitates the gold nanoparticles’ nucleation and growth [56] obtained by using the protocol from Blidar et al., 2022 slightly medicated. Briefly, a constant potential of −0.4 V for 1200 s was applied and an ordered layer of gold nanostructures was formed, creating an Au@SCPE surface. The thickness, calculated from the charge transferred, was 26.54 ng (RSD ± 0.25%, n = 20). The SEM images of pristine SCPE (Figure 4A) are compared with the gold-modified electrode without any carbonated-based pre-treatment (Figure 4B) and after activation (Figure 4C). It is evident that the latter is characterized by a homogeneous dispersion both in size and distribution of the Au nanoparticles and the magnifications (insert in Figure 4A,B) confirm the effective role of the carbonate pre-treatment.

Energy-dispersive X-ray spectroscopy (EDXS) (Table 2) also confirmed a slight increase in the composition of oxygen at the electrodic surface activated. Moreover, even though the gold electrodeposition was apparently higher for the bare graphite electrode than for the electrode treated (lines 3 and 4 in Table 2), the latter, apart from the more uniform distribution, demonstrated enhanced nanoparticle stability after washing (lines 6 and 4 in Table 2). Instead of the 53% leaching measured for the bare graphite electrode, 8% was estimated for the electrode treated in carbonate solution. Gold-modified graphite SPCEs were selected as a cost-effective and scalable alternative to commercial gold electrodes, enabling custom surface engineering. The electrochemical deposition of gold generates a nanostructured morphology that facilitates stable and oriented immobilization of thiolated aptamers through thiol–gold chemistry. Similar strategies based on Au-modified carbon SPCEs have been widely reported for thiolated aptasensor fabrication ([57,58,59,60]).

Preliminary gel electrophoresis experiments (see details in SI) were conducted to test the hybridization between each aptamer and the pertinent capture probes. These tests allowed us to observe the appearance of bands with reduced electrophoretic mobility, compared to the individual components, confirming the formation of stable aptamer–probe constructs. While a difference in band intensity suggested that the hybridization efficiency, a key factor influencing biosensor performance, emerged to be variable through the different “Apt-capture probe” combos tested.

Capture probes, P63_8 and P63_16, were tested in conjunction with the full-length aptamer OA63 to examine conformational changes upon anchoring to the transducer surface [52]. This was carried out using a heterogeneous immobilization strategy where hybridization occurred directly on the electrode surface as per Protocol 1 in Scheme 1, paragraph 2.4.2. The Differential Pulse Voltammetry (DPV) yielded inconsistent results, irrespective of probe length (i.e., anodic: 0.18 μA, cathodic: −0.11 μA at ±0.4 V; CV 64% n = 20) (data not shown). These inconsistencies were attributed to electrostatic interference from the negatively charged gold surface, which likely hindered effective aptamer hybridization [52,53]. To overcome surface interference effects, a solution-phase hybridization strategy was developed (Protocol 2). Pre-formed “aptamer-probe” complexes were subsequently immobilized on the Au@SPCE surface, resulting in reproducible DPV measurements (cathodic: −1.9 μA; anodic: +2.2 μA at ±0.4 V, n = 10). Table 3 summarizes these results, indicating that the most efficient aptamer–probe pairings for OA 63 were with its shorter capture probe P63_8.

To further improve the binding, the thiol activation was performed by comparing TCEP and DTT treatments. The TCEP (250 μM TCEP:2.5 μM aptamer, 45 °C overnight) needed an MCH blocking (1 mM, 60 min, 25 °C) step, as DTT excess-based protocol enabled simultaneous activation and blocking of the electrodic surface. Overall, the DTT approach demonstrated superior reproducibility [61,62], and when combined with the homogeneous immobilization strategy, revealed optimal aptamer–probe pairings. This protocol was applied to OA31 as well, which performed better with its longer capture probe P31_14 (Table 3). These combinations maintained consistent performance across various hybridization times (10–60 min), with 30 min established as optimal for subsequent analyses (Supplementary Materials Table S2) [61,62].

The structural characteristics of aptamer–probe complexes proved critical to sensor performance. Detailed investigation revealed that capture probe length significantly influenced the three-dimensional conformation of the aptamer–probe complex in solution and, consequently, OA binding efficacy. For both aptamer variants, the incorporation of thymine residues within capture probes markedly modulated binding affinity. Previous studies have established that stem length critically affects hairpin conformational dynamics, where suboptimal lengths either prevent conformational changes or result in excessive structural flexibility [63,64]. This phenomenon was observed with P63_16, where extended sequence length introduced excessive flexibility compared to P63_8, resulting in decreased OA63 binding affinity. Conversely, the shorter P31_6 probe’s limited complementarity (six bases) restricted access to OA31 binding sites, yielding substantially reduced binding activity relative to P31_14. The optimal probe lengths (P63_8 and P31_14) appeared to facilitate favorable aptamer folding conformations, as suggested by Lubin et al. [65], enabling optimal presentation of binding sites [45,66].

**Figure 5:**
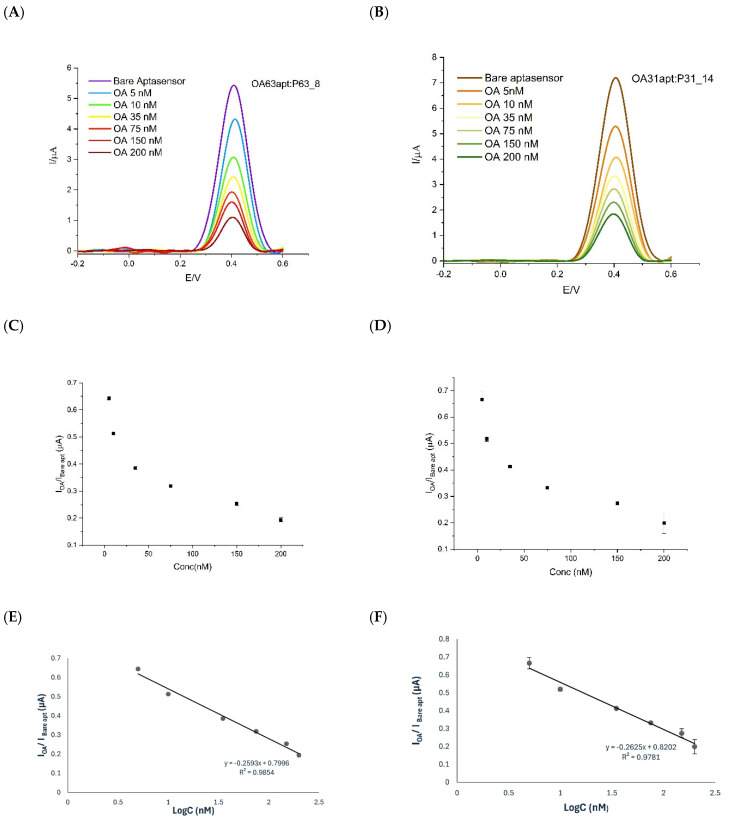
DPV of OA in Tris buffer at the electrode. (**A**) OA63apt + P63_8 probe at 25 °C for 30 min; and for OA in Tris buffer at the electrode. (**B**) OA31apt + P31_14 at 4 °C for 5 min. (**C**) Current ratio (I_OA_/I_Bare apt_) vs. concentration for OA63apt + P63_8. (**D**) Same for OA31apt + P31_14. (**E**) Log-linear calibration plots for OA63apt + P63_8. (**F**) Same for OA31apt + P31_14.

Temperature dependence studies on OA binding by the pertinent Apt, carried out at 25 °C and 4 °C, demonstrated that shorter aptamers exhibited enhanced interaction efficiency at lower temperatures, attributed to increased structural stability and improved target-binding-induced conformational changes [66,67,68]. Conversely, longer aptamer constructs showed superior performance at higher temperatures. Figure 5 illustrates these temperature-dependent binding profiles for both aptamer variants under their respective optimal conditions (Figure 5A,B; all the results of this optimization are available in Supplementary Materials Figure S1). These findings underscore the complex interplay between sequence length, temperature, and binding efficiency in aptamer-based detection systems.

The calibration curves were constructed by plotting the DPV signal of current vs. the logarithm of OA concentration (LogC). Particularly, in the y-axes, to account for the variability of the blank signals, the ratio between the current obtained in the presence of OA bound (I_OA_) vs. the aptamer (I_bare_) incubated only with buffer (control signal) is used. Interestingly, the formation of stable OA–aptamer complexes for EAB OA31apt + P31_14 occurred within 5 min at 4 °C vs. 30 min at 25 °C required by the system OA63apt + P63_8. The 5-min incubation time is notably shorter than those previously reported for similar systems, indicating efficient binding kinetics under optimized conditions even at low temperature. This duration was established through optimization experiments, during which longer incubation times for OA31 did not lead to increased signal intensity. In contrast, such a short incubation period proved inadequate for OA63, which required at least 30 min to reach a stable signal plateau. Thus, the 5-min protocol to bind OA was adopted in the complex OA31apt + P31_14, enabling faster analysis while maintaining sensitivity and reproducibility. The system OA63apt + P63_8 was tested in its optimal condition at 25 °C for 30 min. The log-response (Figure 5C,D) was linearized within the 5–200 nM range investigated with a detection limit of 2.5 nM (calculated as blank signal ± 3σ), for both configurations. The coefficients of variation (CV%) measured ranged from 2 to 10% for both systems while the slight difference in the quality of the linearization (0.73% of difference in the R^2^ value) was compensated for by the speed up of the total analysis (Figure 5E,F). This trade-off between response time and linearity is particularly advantageous for field-based or rapid screening applications.

To better highlight the analytical performance of the proposed aptasensor, Table 4 reports a comparison of the obtained detection limit with those of previously reported electrochemical sensors for okadaic acid.

**Figure 6:**
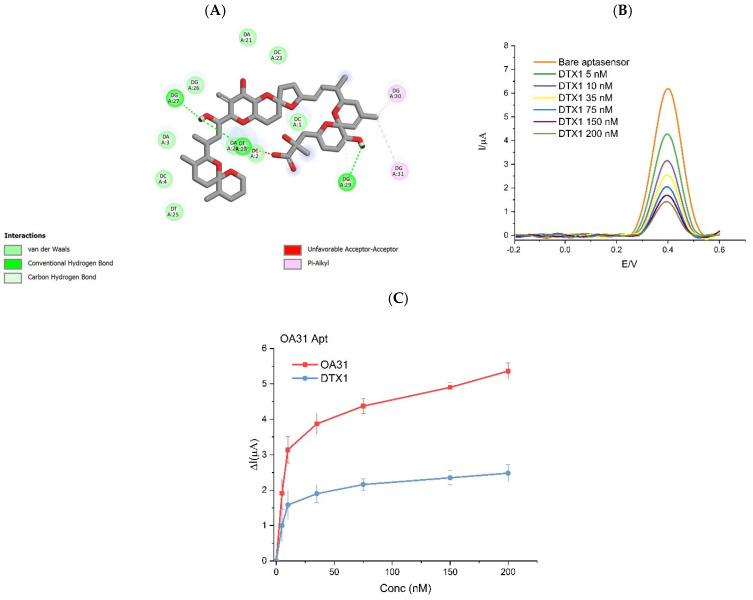
(**A**) Molecular dynamics highlighting the moieties of DTX1 interacting with the aptamer; (**B**) current variation after adding DTX1 to EAB-OA31-P31_14; (**C**) sensitivity of OA31 to OA and DTX1.

The potential cross-reactivity with structurally related dinophysistoxins (DTX1 and DTX2; see Figure 1 in Introduction) was analyzed considering that the structural difference among OA and DTXs is for small substituent groups: DTX1 contains two more methyl (-CH_3_) groups compared to okadaic acid (OA), which only carries hydrogen (-H) groups, whereas DTX2 has a single methyl (-CH_3_) pendant group. To gain preliminary insight into the binding behavior, MD simulations were performed for both DTX1 and DTX2 (details of this investigation are in Supplementary Materials Figure S2) and the 2D interaction diagrams (Figure 6A) confirmed the computational prediction of aptamer binding to both DTX variants.

Since the OA63 aptamer was reported to lack selectivity against structural analogs, experimental validation was only carried out for the new OA31. Moreover, the only DTX parent toxin tested was DTX1 due to its higher toxicological relevance, as it is known to exhibit higher oral toxicity than DTX2, with a toxic equivalency factor (TEF) of 1.5 versus 0.3 for DTX2 [77]. The experimental trend confirmed the affinity for DTX1 by OA31 even if the sensitivity was 2 times lower.

In Figure 6C, the variation in the current signal obtained by adding increasing concertations of OA to the EAB-OA31Apt is compared with the signal generated by the addition of DTX1. The results show the lower sensitivity of OA 31 toward the parent toxin DTX1.

The practical applicability of E-AB was rigorously evaluated through analysis of complex matrices using spiked mussel samples. The validation protocol involved OA spike-and-recovery studies, with samples equilibrated for 24 h prior to extraction. Following methanol extraction, samples underwent gentle nitrogen evaporation at room temperature before reconstitution of the dry samples in Tris buffer for analysis. The optimized protocol was applied and a recovery rate of 82–103% for OA was calculated even if an increase in background signal was observed, suggesting interferences coming from the complex matrix but minimally pre-treated. Notably, no shift in the peak potential (Ep) of the Fc tag was observed, indicating stability of the electrochemical behavior of the system. These findings indicate that the E-aptasensor shows promising features for real-sample analysis and the detection of DTX1, despite reduced sensitivity, suggesting applicability in total toxicity assessment.

## 4. Conclusions

This investigation advances the domain of marine toxin detection through the development of a rationally designed electrochemical aptasensor for okadaic acid (OA) by computational predictions. The first evidence is the confirmation of the fundamental relationship between aptamer and capture probe architectures affecting the functioning of EAB. Specifically, the optimal performance of the full-length OA63 aptamer was achieved with a shorter capture probe (P63_8), whereas the truncated OA31 variant necessitated a longer, thymine-rich probe (P31_14), confirming the need of both mobility and proper distance from the electrodic surface to gain binding of the target.

Molecular docking simulations were instrumental in guiding the design and truncation of the OA63 aptamer sequence, furnishing initial insights into critical binding regions and potential selectivity profiles. However, our findings underscore the need for rigorous experimental validation to critically verify in silico predictions. Such computational models, by their inherent nature, may overlook crucial experimental variables, including temperature, incubation time, and the steric and mobility constraints imposed by aptamer immobilization on the electrode surface.

Regarding selectivity, docking simulations suggested that the truncated OA31 aptamer would bind both OA and DTX1, indicating a degree of cross-reactivity attributed to the absence of counter-selection during the original SELEX process and the simplified environment of the docking simulation. Experimental validation corroborated this prediction, demonstrating a response to DTX1, albeit with significantly lower sensitivity compared to OA. This outcome, while supporting general binding trends from docking, concurrently highlights the inherent limitations of computational modeling in precisely forecasting fine selectivity under complex, real-world conditions.

Despite these challenges, our study demonstrates the efficacy of in silico truncation as a viable strategy for aptamer optimization. The shortened OA31 sequence successfully retained effective target recognition, thereby validating its utility in rational aptasensor design. While our achieved detection limit of 2.5 nM may not surpass the ultra-low sensitivities reported by certain other systems (e.g., Eissa et al., 2013: 0.18 nM; Gu et al., 2016: 0.025 nM) [42,75], our platform offers distinct advantages in practical application. These include remarkably rapid detection times of 5 min compared to traditional assay times generally ranging from 30 to 60 min, and minimal sample pre-treatment, features that render the optimized EAB here well-suited for on-site monitoring applications where speed and simplicity are paramount.

In comparison with earlier electrochemical aptasensors for OA (e.g., [43]), which were mainly developed through empirical optimization of the aptamer–probe interface, our work introduces a rationally designed workflow that integrates in silico docking with electrochemical validation. This dual approach streamlined the identification of an efficient truncated aptamer and its optimal complementary probe, while also anticipating selectivity issues and providing a deeper understanding of the process than conventional trial-and-error methods.

Taken together, this work provides a realistic perspective on integrating molecular modeling into aptasensor development. While computational docking significantly accelerates sequence screening, this study reaffirms that experimental validation is indispensable for navigating real-world complexities. The resulting platform achieves a functional balance between analytical speed and sensitivity, a critical trade-off inherent in portable biosensor design. To further enhance performance, future work should focus on overcoming this compromise. For instance, multi-aptamer arrays could be employed to improve sensitivity and specificity, while implementing counter-SELEX would refine aptamer selectivity against key interferents. Moreover, machine learning-guided design offers a powerful route to discover novel sequences with superior binding kinetics from the outset [78,79,80,81,82]. By building on the insights from this study, these advanced strategies can guide the development of more robust and sophisticated systems for environmental toxin monitoring.

## Figures and Tables

**Table 1:** Structure of the designed aptamers and capture probes.

**Aptamers**
**-OA63**: ((Fc)5′-CCG GGT GGG TGG GTG TGG TCT TGT ATT TGA TTA TGT CTG TCG GCG CTT TTT GGC CCC TTC GTT-3′) **-OA31**: (5′-CCA CCA ACG AGA GTC AGA AAA CCA TGG TGG G-3′(Fc)).
**Capture probes**
**P63-8**: ((SH)5′-AACGAAGG-3′) **P63-16**: ((SH)5′-TTTTTTTTAAGCAAGG-3′) **P31_6**: (5′-TGGTGG-3′(SH)) **P31_14**: (5′-TGGTGGTTTTTTTT-3′(SH))

**Table 2:** Composition (%) of the graphite electrode measured by EDXS when used as such or after pre-treatment made with NaHCO_3_.

Sample	C	O	S	Cl	Au
(1) Bare Graphite (**BG**)	9.323 ± 0.151	2.003 ± 0.144	0.067 ± 0.006	2.610 ± 0.026	-
(2) Graphite pre-treated NaHCO_3_ (**PT**)	94.883 ± 0.331	3.593 ± 0.055	0.073 ± 0.015	2.450 ± 0.338	-
(3) AU at BG	86.800 ± 4.456	1.413 ± 0.255	0.430 ± 0.140	3.430 ± 0.164	7.927 ± 4.031
(4) AU at PT	89.390 ± 2.340	1.390 ± 0.201	0.377 ± 0.99	2.953 ± 0.324	5.887 ± 1.892
(5) AU at BG after washing	91.623 ± 2.588	1.360 ± 0.151	-	3.260 ± 0.397	3.757 ± 2.370
(6) AU at PT after washing	89.557 ± 2.069	1.530 ± 0.337	0.083 ± 0.144	3.433 ± 0.095	5.397 ± 1.978

**Table 3:** Comparison of the capture probe length effect in the immobilization of the complex “probe-aptamer” at the electrode surface. The signal is the anodic current (μA) measured by DPV due to the ferrocene (Fc) redox probe tagging the aptamers.

	Probe Length	P63_8	P63_16	P31_6	P31_14
Aptamer-Fc					
**OA63**		5.27 μA (n = 10, ±8%)	3.59 μA (n = 10, ±18%)		
**OA31**				4.31 (*n* = 10, ±23%)	6.83 μA (*n* = 10, ±5.50%)

**Table 4:** Comparison of analytical methods and aptasensor for okadaic acid detection.

Technique	LOD (nM)	Assay time	Matrix	Ref.
HPLC-FLD	18.63 nM	2–3 h	Mussel extracts	[69]
LC–MS/MS	0.25–6 nM	3–4 h	Mussel extracts	[70,71]
ELISA/Immunoassays	0.0057–18 nM	~1 h	Mussel extracts	[21,72,73]
PPIA	0.16–1.8 nM	~1 h	Mussel extracts	[74]
Aptasensor	0.0001–8 nM	20 min–~1 h	Buffer solution, (PBS), Mussels extracts	[42,45,75,76]
This work	2.5	5 min	Mussel extracts	—

## Data Availability

Data are contained within the article and Supplementary Materials.

## Supplementary Materials

The following supporting information can be downloaded at: https://www.mdpi.com/article/10.3390/bios15100665/s1, Figure S1: Test for all combinations of capture probes and aptamers test (a) OA63-P63_8 4 °C OA recognition complex and calibration curve, (b) OA63-P63_16 25 °C OA recognition complex and calibration curve, (c) OA31-P31_6 OA recognition complex and calibration curve 4 °C, (d) OA31-P31_14 25 °C complex OA recognition complex and calibration curve; Figure S2: 3D rearrangements and 2D moieties involved in (A) OA31 and DTX1, (B) OA31 DTX2, (C) OA63 and DTX1, (D) OA63 and DTX2 binding; Table S1: Parameters optimised to perform the measurements; Table S2: Effect of the hybridation time in solution for OA63 and P63_8 and subsequent immobilization overnight at 4 °C at AuNP@GE.
