# Supplementary material for: Harnessing in Silico Design for Electrochemical Aptasensor Optimization: Detection of Okadaic Acid (OA)
Source: Biosensors (Basel). 2025 Oct 3;15(10):665. doi: 10.3390/bios15100665 (PMC12562321; doi:10.3390/bios15100665)
Supplement: Supplementary file 1 [file biosensors-15-00665-s001.zip › biosensors-3844138-supplementary.pdf]

Harnessing in Silico Design for Electrochemical Aptasensor Optimization: Detection of Okadaic Acid (OA)

Margherita Vit [1], Sondes Ben-Aissa [2], Alfredo Rondinella [3], Lorenzo Fedrizzi [3] and Sabina Susmel [1,*]

1. Bioanalytical Chemistry and Biosensors Lab, Department of Agri-Food, Environment and Animal Sciences (Di4A), University of Udine, Via Sondrio 2/A, 33100 Udine, Italy; margherita.vit@uniud.it

2. Molecular Sciences Research Hub, Department of Chemistry, Imperial College London, London W12 0BZ, UK; s.ben-aissa@imperial.ac.uk

3. Polytechnic Department of Engineering and Architecture, University of Udine, Via del Cotonifico 108, 33100 Udine, Italy; alfredo.rondinella@uniud.it (A.R.); lorenzo.fedrizzi@uniud.it (L.F.)

*Correspondence: sabina.susmel@uniud.it

1. Electrochemical measurements and optimal amount of gold electrodeposited at the SPCE

The electrodeposition of gold to form AuNPs at graphite electrode was tested by using different electrochemical approaches summarised in SI_Table S1. Cyclic voltammetry sweeps linearly over time the voltage between 2 voltage limits as in amperometry the proper voltage for electroreduction is applied over a finite time-window.

SI Table S1: Parameters optimised to perform the measurements

Technique	Ei (initial voltage V^{-1})	Ef (final voltage V^{-1})	Scan rate (mV sec^{-1})	Time/s
Cyclic voltammetry (CV)	-0.8	0.8	0.1	ND
Differential Pulse Voltammetry (DPV)	-0.5	0.6	0.1	ND
Amperometry	-0.4	ND	ND	1200

2. Gold Thickness from the charge transferred

The AuNPs are forming after reduction of Au (III) to Au (0) accordingly to eq. 1

$$AuCl_4^- \ (aq) + 3e^- \ \rightarrow Au(s) + 4Cl^- \qquad \text{eq.1}$$

Chronomaperometry produced a gold layer coverage more reliable and stable to the washing step and the amount of gold deposited was estimated by applying the Faradays laws. The flow of current through an electrolyte (i.e., mass of chemical deposition) is directly proportional to the charge (coulombs) passed through it (Eq. 2.1). Moreover, the mass of a substance deposited at any electrode passing a certain amount of charge is directly proportional to its chemical equivalent weight (Eq. 2.2), and finally, by multiplying m_{eq} for the Faraday (F) corresponding to the charge deposited.

$$m = QZ$$

Equation 2.1 First law of Faraday

Where: Q is the charge (in coulombs or Ampere seconds),

Z is the electrochemical equivalent (i.e eq) or mass of the substance deposited per unit charge

$$m_{eq} = \frac{m_m}{n_{e-}}\left(\frac{g}{eq}\right)$$

Equation 2.2 Second law of Faraday

Where: m_{eq} is mass of a substance deposited at any electrode

m_m is the molar weight of the substance undergoing the redox process

n_{e-} is the valency value of the element

3. Protocol for the Electrophoresis on agarose gel

To preliminary test the affinity between each apt and the pertinent capture probes, a 2.5% (w/w) of agarose gel in Tris-Borate-EDTA (TBE) was prepared by heating the solution until boiling, then it was poured on a tray, letting it cool down until a firm gel was formed. A solution of each probe and aptamer in ratio 1 to 1 (1.5 µM) was heated up to 95°C for several minutes (10, 30 and 60 min , respectively) to let the two single stranded DNAs (ssDNAs) react and hybridize. After cooling, a volume of 20µL of each solution was loaded into the sample wells, alongside with a loading gel and ethidium bromide (EtBr) used strictly respecting the rules for safe handling and dispose. After a 45 minutes running at 60V the gel was observed under the UV lamp.

4. Optimisation of the time of hybridisation carried out in solution at 90°C

The aptamer and its capture probe were hybridised in solution before to place the complex in contact with the electrode surface modified by gold electrodeposition. The incubation carried out in buffer at 90°C was tested at 3 different time, namely 10, 30, 60 min. Finally, the optimal condition was 30 min.

SI Table S2. Effect of the hybridation time in solution for OA63 and P63_8 and subsequent immobilization overnight at 4°C at AuNP@GE

	**Time @ 90°C**		
**Current (µA)**	**10 min**	**30 min**	**60 min**
Anodic	2.8 µA (± 12 %)	5.27µA (± 8 %)	2.2 µA (± 12%)
Cathodic	-2.3 µA (± 13 %)	-5.12 µA (± 10 %)	-1.9 µA (± 14 %)

5. Optimisation of the EAB

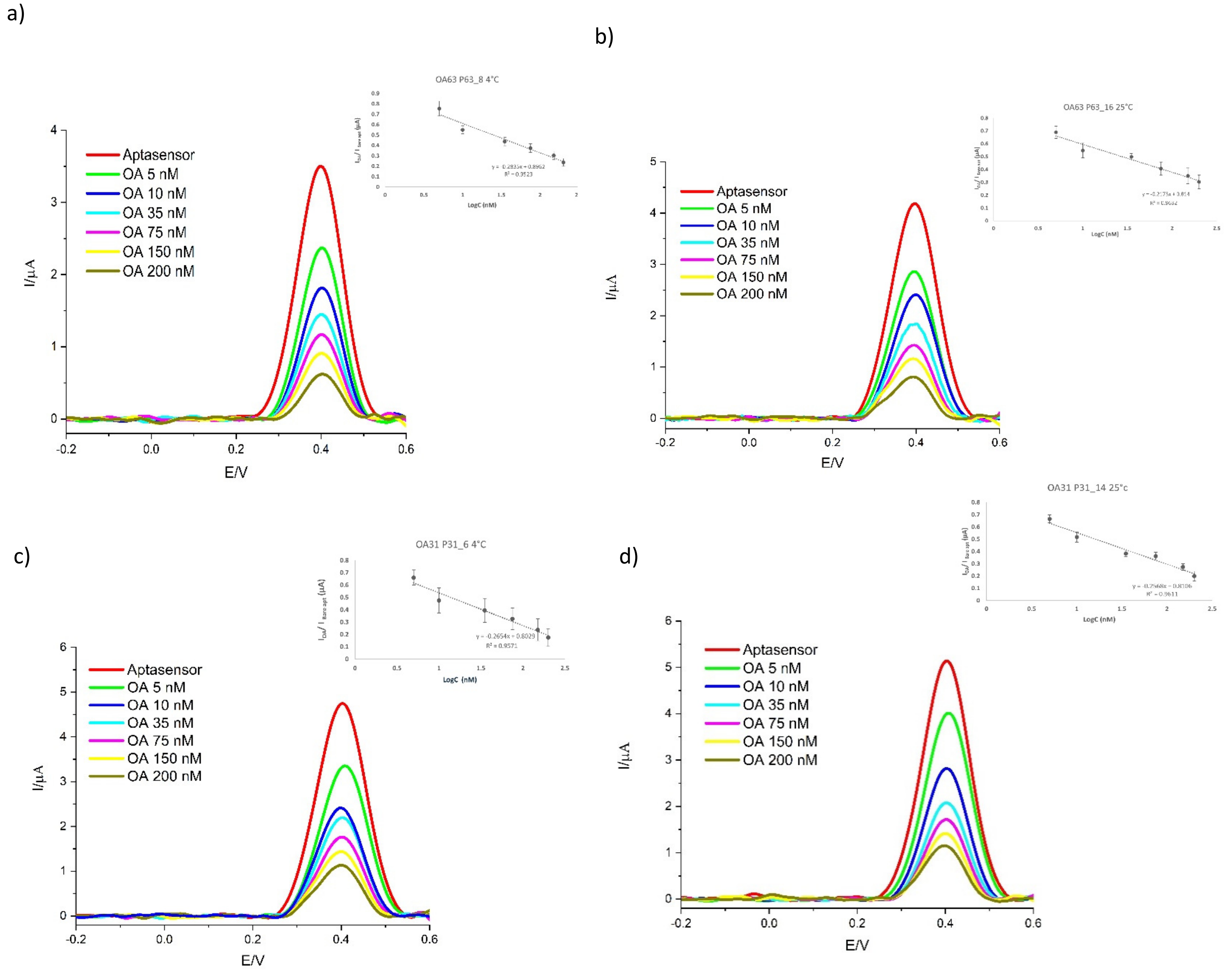

SI Figure S1: Test for all combinations of capture probes and aptamers test a) OA63-P63_8 4°C OA recognition complex and calibration curve, b) OA63-P63_16 25°C OA recognition complex and calibration curve , c) OA31-P31_6 OA recognition complex and calibration curve 4°C d) OA31-P31_14 25°C complex OA recognition complex and calibration curve

6. *In silico* investigation of the interactions Apts - parents toxins DTX1 and DTX2

All selectivity MD simulations with OA31 and OA63 toward DTX1 and DTX2

a) OA31 and DTX1

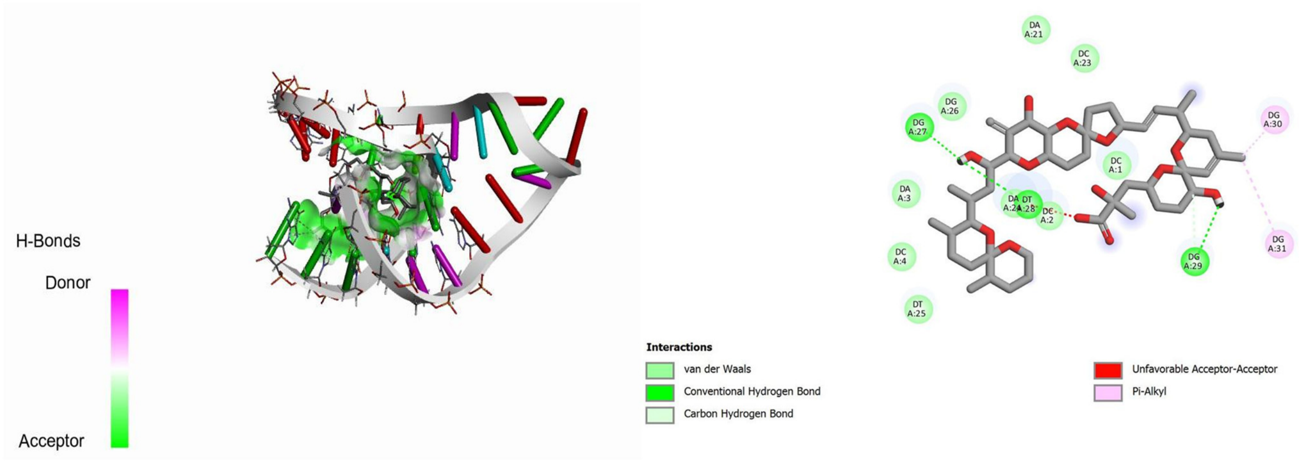

b) OA31 DTX2

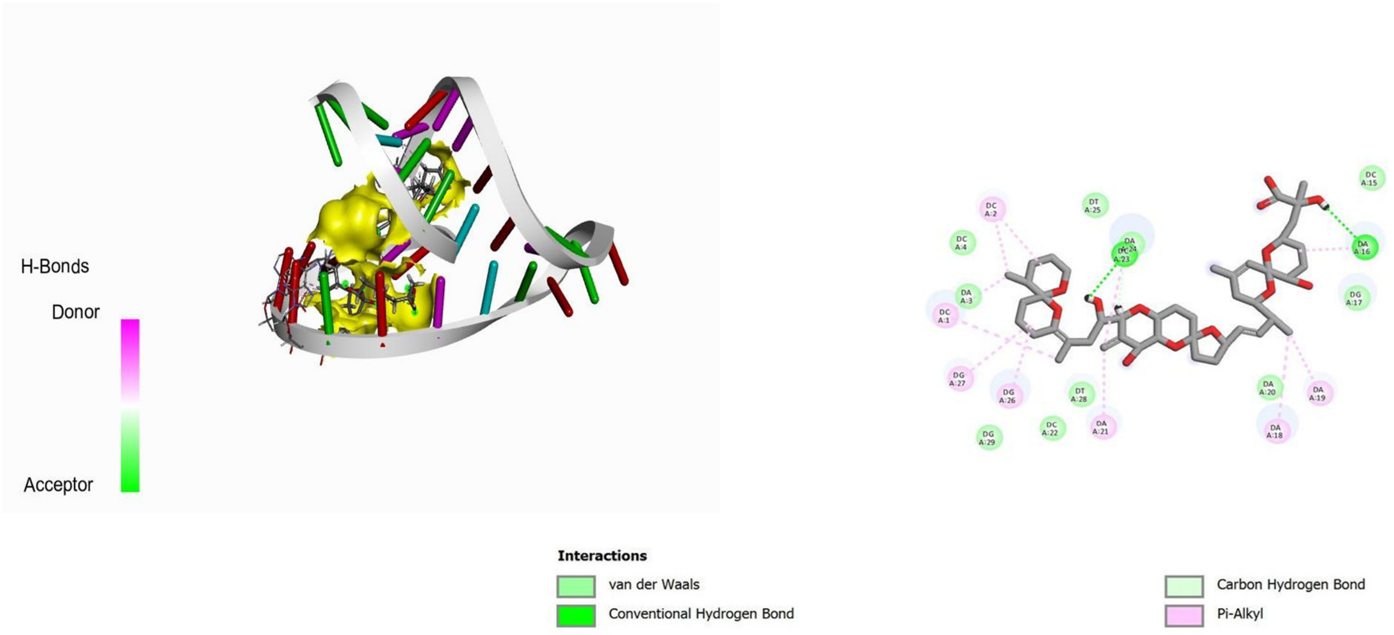

c) OA63 and DTX1

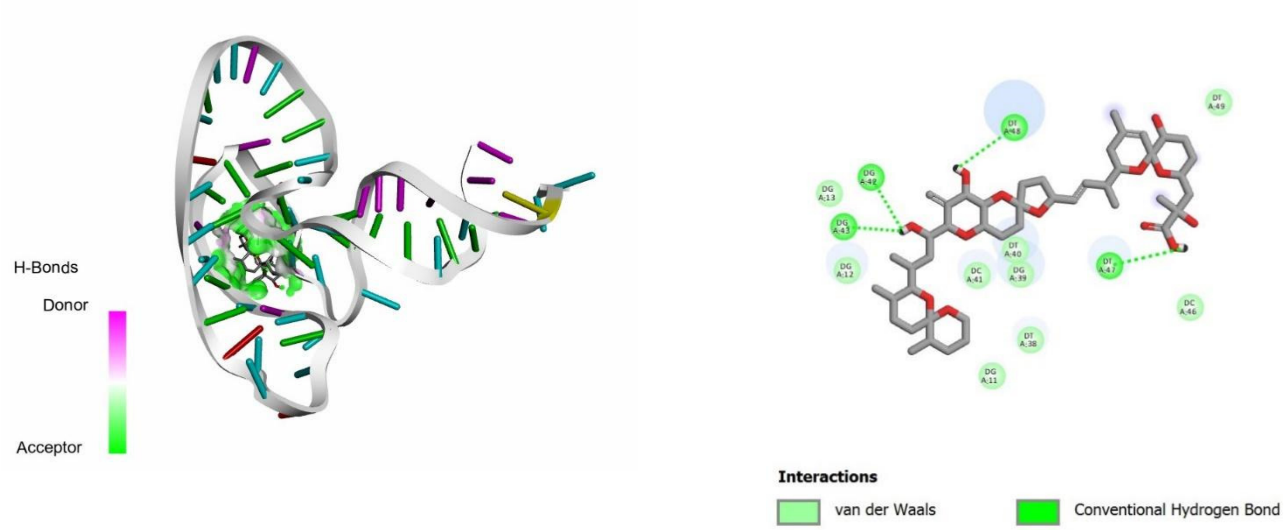

d) OA63 and DTX2

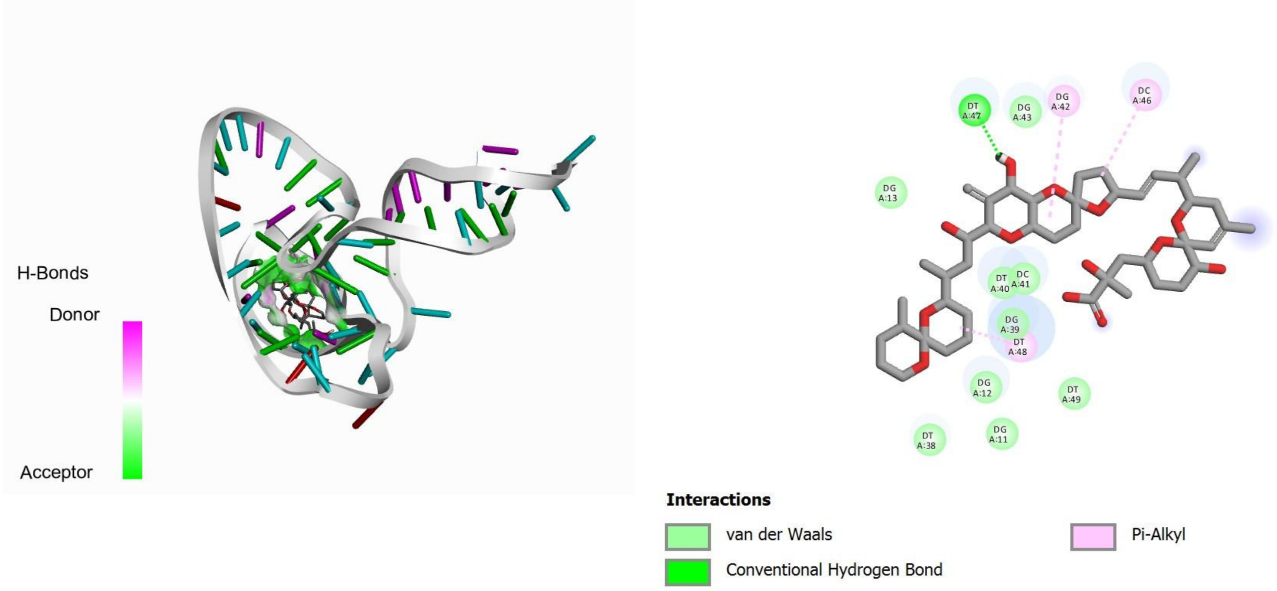

SI Figure S2: 3D rearrangements and 2D moieties involved in A) OA31 and DTX1, B) OA31 DTX2, C) OA63 and DTX1, D) OA63 and DTX2 binding
